# Dataset on species incidence, species richness and forest characteristics in a Danish protected area

**DOI:** 10.1016/j.dib.2016.10.021

**Published:** 2016-11-01

**Authors:** Adriano Mazziotta, Jacob Heilmann-Clausen, Hans Henrik Bruun, Örjan Fritz, Erik Aude, Anders P. Tøttrup

**Affiliations:** a Center for Macroecology Evolution and Climate, Natural History Museum of Denmark, University of Copenhagen, Copenhagen, Denmark; b Section for Ecology and Evolution, Department of Biology, University of Copenhagen, Copenhagen, Denmark; c Naturcentrum AB, Sweden; d HabitatVision A/S, Denmark

**Keywords:** Bryophytes, Forest restoration, Lichens, Space-for-time substitution, Vascular plants, Wood-inhabiting fungi

## Abstract

The data presented in this article are related to the research article entitled “Restoring hydrology and old-growth structures in a former production forest: Modelling the long-term effects on biodiversity” (A. Mazziotta, J. Heilmann-Clausen, H. H.Bruun, Ö. Fritz, E. Aude, A.P. Tøttrup) [1]. This article describes how the changes induced by restoration actions in forest hydrology and structure alter the biodiversity value of a Danish forest reserve. The field dataset is made publicly available to enable critical or extended analyses.

**Specifications Table**

**Table:** 

**Subject area**	Biology
**More specific subject area**	Forest Ecology and Management, Restoration, Conservation Biology
**Type of data**	Excel file
**How data was acquired**	Procedures of collection described in Data collection, paragraph 2.3. in Materials and methods of Mazziotta et al. [1].
**Data format**	Raw, Analyzed
**Experimental factors**	Information for each variable related with species richness and forest characteristics were aggregated at plot level.
**Experimental features**	The relationships between the species richness of four ecological groups and their associated habitat characteristics were determined
**Data source location**	Lille Vildmose nature reserve, Denmark (56°509 N, 10°159 E)
**Data accessibility**	The data are available within this article

**Value of the data**

• This data can be used to compare the structures of species communities in other temperate forests.
• This data helps to compare the effect of environmental drivers of species richness in other temperate forests.
• This data can be used as training data in modelling exercises in statistical classes.

## 1 Data

In this article׳s data file in Supplementary material, “DiB_A.xls”, we stored 5 excel sheets:

– In the matrices of the first four excel sheets (i.e. “bryophytes”, “lichens”, “fungi”, “plants”) we recorded species (species Latin name in Column A) incidence (presence–absence) for each ecological group analyzed in the article (i.e. epiphytic bryophytes, epiphytic lichens, vascular plants and wood-inhabiting fungi) in each of the 102 forest plots object of the 2013 monitoring campaign in the Lille Vildmose forest reserve (Denmark) (columns B–CY).
– In the last excel sheet (Table “Richness_Env” in DiB_A.xls) we recorded plot coordinates (columns A–B) and plot number (Column C), along with the forest area they belong to (Column D), the species richness of each of the 4 ecological groups analyzed (columns E–H), the values of the predictor variables used to model species richness: basal area (X_BA) (columns I–M) and deadwood (X_DW) (columns N–P) for each of the tree genera (X); Number of Trees with Rotten Parts (NTRP, column Q), Stand Age (SA, column R), number of tree genera (Tree, column S), measured water level in 2013 (WL2013, column T) and predicted water level by 2050 (WL2050, column U).

## 2 Experimental design, materials and methods

In the study area of Lille Vildmose we sampled species and forest characteristics in 102 circular sample plots (radius 15 m), settled at a minimum distance of 30 m and with a 15 m minimum buffer from forest edges. In each of the 102 forest plots, we recorded species presence-absence for each ecological group, i.e. vascular plants, wood-inhabiting fungi, and epiphytic bryophytes and lichens with the following procedures during 2013:

– Vascular plants were recorded in a 5 m circular plot concentric with the study plot. Vegetation was monitored in summer;
– Fruit bodies of wood-inhabiting fungi were recorded per each deadwood item in the plot in August and October);
– Epiphytic bryophytes and lichens were recorded on (1) standing live and dead trees with DBH ≥ 10 cm (from 0 up to 2 m height) and on (2) stumps taller than 1.5 m; epiphytes were surveyed in April, August and October.

Species identification in all the surveys was carried out in the field if possible, otherwise with the help of a microscope in the lab.

The following variables were measured across sample plots during the species sampling in 2013, mainly following the standard protocols given in [2]: 1) dimensions and tree species for all deadwood items with diameter ≥ 10 cm and length ≥ 1 m; 2) all living trees with diameter ≥ 10 cm identified and their DBH measured; 3) number of trees with rotten parts; 4) water level (WL) averaged from 4 different measures, each taken 5 m from the center of the plot. If WL was above ground, water surface depth was considered as a positive value. Otherwise, a small hole was dug and distance from the ground level to the raised water surface was considered as negative. If no water rose when 40 cm depth was reached, the value was recorded as “>−40 cm”. Maximum WLs were measured for all the plots on 24th -25th February 2013. Future WLs by 2050 were estimated by means of hydrological models (described in [3], [4]); 5) stand age for each plot was taken from the forestry maps of the area.
